# Clinical decision-support for acute burn referral and triage at specialized centres – Contribution from routine and digital health tools

**DOI:** 10.1080/16549716.2022.2067389

**Published:** 2022-06-28

**Authors:** Constance Boissin

**Affiliations:** Department of Global Public Health, Karolinska Institutet, Stockholm, Sweden

**Keywords:** Clinical decision-making, injuries, computer-assisted diagnosis, emergency service, accident prevention

## Abstract

**Background:**

Specialized care is crucial for severe burn injuries whereas minor burns should be handled at point-of-care. Misdiagnosis is common which leads to overburdening the system and to a lack of treatment for others due to resources shortage.

**Objectives:**

The overarching aim was to evaluate four decision-support tools for diagnosis, referral, and triage of acute burns injuries in South Africa and Sweden: referral criteria, mortality prediction scores, image-based remote consultation and automated diagnosis.

**Methods:**

Study I retrospectively assessed adherence to referral criteria of 1165 patients admitted to the paediatric burns centre of the Western Cape of South Africa. Study II assessed mortality prediction of 372 patients admitted to the adults burns centre by evaluating an existing score (ABSI), and by using logistic regression. In study III, an online survey was used to assess the diagnostic accuracy of burn experts’ image-based estimations using their smartphone or tablet. In study IV, two deep-learning algorithms were developed using 1105 acute burn images in order to identify the burn, and to classify burn depth.

**Results:**

Adherence to referral criteria was of 93.4%, and the age and severity criteria were associated with patient care. In adults, the ABSI score was a good predictor of mortality which affected a fifth of the patients and which was associated with gender, burn size and referral status. Experts were able to diagnose burn size, and burn depth using handheld devices. Finally, both a wound identifier and a depth classifier algorithm could be developed with relatively high accuracy.

**Conclusions:**

Altogether the findings inform on the use of four tools along the care trajectory of patients with acute burns by assisting with the diagnosis, referral and triage from point-of-care to burns centres. This will assist with reducing inequities by improving access to the most appropriate care for patients.

## Background

### Acute burn diagnosis – a difficult task

Globally, poor access to care in general and to specialized care in particular impact negatively on morbidity and mortality, not least in the case of injuries [1,2]. Burn injuries for instance, the fourth-largest cause of injury death worldwide with approximately 418 deaths a day, challenge health-care systems [3]. They are difficult to assess accurately at point-of-care, triage mechanisms are not in place everywhere, and specialized centres are few and overloaded. While small burns should be treated locally and severe ones transferred to specialised centres, overtriage leads to the transfer of futile cases (‘those in whom goals of care cannot be met at any time’ [4]) that overburdens unduly the system [5].

A burn’s severity is defined by its size – measured as the total body surface area (TBSA) affected – and its depth. Depth is split into five anatomic categories (superficial thickness, superficial-partial thickness, mid-partial thickness, deep-partial thickness and full thickness [6]), that can be further divided into two surgical treatment strategies, namely partial injuries (that include superficial-partial to mid-partial thickness burns and which will heal spontaneously) and deep injuries (that include deep-partial thickness and full thickness burns that will require surgical intervention and skin grafting in order for them to heal) [7,8]. Burn size and depth are most commonly estimated through visual and tactile assessments, but misdiagnoses are frequent for both, as observed in emergency centres and among referring physicians, and, for burn depth, even among surgeons [9–16].

Decision support aids exist to help minimize referral and triage errors. Some are more traditional, list-based and are meant to be used at bedside and others – digital – provide remote assistance. Four such tools are covered in this thesis synthesis, two that are used at admission to specialised burns centres and the other two as a means to provide assistance at point-of-care.

### Existing decision-support tools at admission to burns centres

Triage is the sorting of patients for treatment in situation of at least modest resource scarcity, according to an assessment of the patient’s medical condition and the application of an established sorting system or plan [17]. Burn triage can be context-specific, referring to patient selection for referral, admission or discharge when resources are scarce [18–20], or solely to patient (air) transport, when resources are less of an issue [21–24].

Referral guidelines exist to define which conditions should be referred to burns centres. A first version was developed by the American Burn Association [25,26], and then adapted to several contexts including in Australia and New Zealand [27], in Europe [28], or in South Africa [29]. While several criteria are similar across guidelines e.g. the involvement of sensitive body parts, others are dependent on the availability of resources, such as the size of the burn at which a patient should be transferred. How well guidelines are implemented has been documented only for a number of emergency or referring centres [30–36] and burns centres [21–23,37–43]. The bulk of these studies are however from high-income settings where the lack of beds in burns centres is not as big and constant issue.

In addition to referral guidelines, mortality prediction scores – based on key patient and injury factors – can also aid with patient flow by identifying futile patients and those likely to survive [44]. The limited resources can then be used in priority for those who would benefit the most from them [20,45–47]. Factors identified in different patient populations include age, burn size, presence of inhalational injury, gender, presence of comorbidities, and burn depth [48,49]. The Abbreviated Burns Severity Index (ABSI) score is one of the existing prediction scores in which the risk of mortality is obtained by adding assigned scores to five predictive variables observed at admission: sex, age, inhalation injury, burn depth, and TBSA [50]. This score has been used in a number of settings, including in Switzerland, Indonesia, Malaysia and Ghana [51–55], while in others such as in a Kenyan and in a South African burns unit the original score had to be modified in order for mortality breakpoints to represent the studied populations [56,57].

### Newly-developed digital tools: an opportunity for remote diagnosis

Although the ultimate triage tool would be based on easily identifiable and interpretable injury characteristics, it will always be reliant on the initial diagnosis which should be as accurate as possible. In recent years, the dramatic increase in smartphone’s and tablet’s penetration in all settings has brought a fantastic opportunity for digital health applications [58] with the potential to remove existing distances between patients and specialists and to provide experts, advice directly at point-of-care [59–65]. In fact, the visual nature of burn injuries makes them a perfect candidate for using such image-based technologies. Several spontaneous expert-led informal [66,67] and formal [68] initiatives for the transfer of information and knowledge have been implemented in the past few years and are even recommended prior to referral in some countries such as France or the USA [25,69]. These latter recommendations however rely on the viewing of images on specifically designated computers [62], a method that has been proven for a number of years [70–73]. This is nonetheless unrealistic in settings where handheld devices are much more common than computers. Whether these devices provide an accurate diagnosis needs to be investigated in order to verify that all patients have access to appropriate and equitable treatment independently of the context.

Remote consultation will however always be limited by the small number of burns specialists who cannot assess all cases [74]. The recent development in automated image analysis using deep-learning algorithms could be a potential solution to alleviate this problem [75]. In particular, convolutional neural networks (CNNs) have been used to assist with diagnosis in other medical disciplines such as in dermatology [76], ophthalmology [77], radiology [78], pathology [79], or cardiology [80]. In burn care, automated burn size calculations have been suggested by the use of several mobile apps [81–83]. A few additional studies have investigated the potential of deep-learning to distinguish small areas of burns from other wounds [84, 85] or healthy skin [84–86]. Only one study has developed an algorithm for the determination of burn depth using 23 images and obtaining an accuracy of approximately 80% [87]. Like for other CNN-based medical studies, there is a bias in the existing body of evidence, where only one study currently involves patients with different skin types, and which concluded on the complexity of training a combined model for all skin types [84].

The overarching aim of this work was to synthesize the doctoral thesis defended at Karolinska Institutet, Sweden. The aim of this thesis was to determine the potential of decision-support tools for referral and triage of acute burns injuries by assessing four of them used in South Africa and Sweden: referral criteria, mortality prediction scores, image-based remote consultation and automated diagnosis. The following research questions were addressed, each in one sub-study

- What is the level of adherence to existing referral criteria at admission and to what extent are each of these criteria associated with the care received at a paediatric burns centre? (Study I [88],)
- What are the determinants associated with in-hospital mortality, and to what extent could the ABSI score be used as a source of triage at admission to an adult burns centre? (Study II [89],)
- How accurate is the image-based remote diagnosis of burns when viewed on a handheld device? (Study III [90],)
- What is the accuracy of an image-based deep learning algorithm for identifying a burn wound, and classifying burn depth? How are those results affected by skin types of patients? (Study IV [91],)

## Methods

### Evaluation of existing triage tools at specialized burns centres (Studies I and II)

#### Setting

Studies one and two took place in the Western Cape Province of South Africa, which is the third largest province in the country both in terms of land and population [92,93]. The province’s demography is marked by a high immigrant rate with an observed increase population size of 20% in the last ten years [93]. This population is vulnerable, with one in five inhabitants living in informal housing [94], some not having access to electricity for lighting or cooking [92].

The healthcare system in South Africa is two-tiered and it is estimated that 84% of the South Africans are users of the publicly funded system, leading to high inequities in access and quality of care [95,96]. The public healthcare system in the Western Cape consists of 435 primary healthcare clinics, 34 district hospitals, five regional hospitals and three tertiary hospitals [97]. However, in this context burn care has to rival against a quadruple burden of disease which includes maternal and child mortality, infectious diseases, non-communicable diseases and violence [98]. There are only two burns centres in the province, both located in the city of Cape Town, together consisting of 46 beds and managing approximately 1950 patients annually [99]. The paediatric burns centre, located at the Red Cross War Memorial Children’s Hospital (hereafter referred to Red Cross Hospital) does manage a three times higher caseload than the adult burns centre located at Tygerberg Hospital [99]. Burn injuries referral and treatment guidelines have been produced at a provincial level in 2011, and specify that only the most severe and complex burns should be referred and admitted to one of the two burns centres [100]. These criteria are presented in Supplementary Figure 1.

#### Data collection procedure

Patients admitted to both burns centres were identified from the centres’ admission books and their files were then individually retrieved to collect data using a standardized electronic case report form. The forms captured data on the patient (age, and gender), the injury (mechanism, intent, burn depth and size (expressed as percentage TBSA), ABSI score, anatomical site involved, and existing comorbidities), referral (mode of transport to hospital, referring hospital, and adherence to each of the provincial referral criteria), as well as patient management and outcome (admission to the intensive care unit (ICU), treatment, surgery, length of stay and mortality).

#### Samples

Data were collected at the Red Cross Hospital’s paediatric burns centre (Study I) during the busiest times of five consecutive years (winters: May 1^st^- August 15^th^) from 2011 to 2015 and included all patients less than 13 years who presented for acute burns. For Study II, due to the smaller number of patients admitted at the adult burns centre, all patients admitted during full calendar years 2015 and 2016 with acute burns to the adults burns centre at Tygerberg Hospital were included.

A total of 1165 paediatric and 372 adult patients were included in Study I and II respectively. Table 1 presents the patient and injury characteristics by study. Regarding patient outcomes, 28% and 71% of the paediatric and adult cases, respectively, underwent surgery during their stay. In addition, only 26% of the paediatric patients stayed longer than a week in hospital whereas 81% of the adult populations stayed that long. Finally, none of the paediatric patients included in Study I died during their stay, whereas 21% of the adult patients (an even higher proportion of 28% for flame burns) did.

**Table 1.** Patient and injury characteristics of cases included in each of the burns centres, in Study I for paediatric cases at the Red Cross Hospital (n = 1165) and in Study II for adult cases at Tygerberg Hospital (n = 372).

	Paediatric		Adults	
	n	%	n	%
Patient Characteristics				
*Gender*				
Men	649	55.7	250	67.2
Women	516	44.3	122	32.8
*Age group (in years)*				
*<2*	604	51.9	N/A	
*2–3*	312	26.8	N/A	
*4–5*	106	9.1	N/A	
*6–12*	143	12.3	N/A	
*13–20*	N/A		50	13.4
*21–40*	N/A		214	57.5
*41–60*	N/A		95	25.5
*61–90*	N/A		13	3.5
Injury Characteristics				
*Mechanism*				
Hot liquid	953	81.8	72	19.4
Hot object	78	6.7	5	1.3
Fire/Flame	108	9.3	263	70.7
Electrical or Chemical	23	2.0	28	7.5
Unknown	3	0.3	4	1.1
*Anatomical site^a^*				
Head, face and neck	594	51.0	259	69.6
Arms and/or hands	714	61.3	319	85.8
Trunk	605	51.9	250	67.2
Genitalia/Perineum	112	9.6	31	8.3
Legs and/or feet	446	38.3	183	49.2
*Burn size (TBSA, in %)*				
≤5	473	40.6	42	11.3
6–10	372	31.9	52	14.0
11–15	183	15.7	42	11.3
16–30	107	9.2	122	32.8
>30	21	1.8	114	30.6
Unknown	9	0.8	0	0.0
*Burn depth^b^*				
Superficial-partial	1021	87.6	24	6.5
Mid-partial/Indeterminate	15	1.3	106	28.5
Deep-partial	N/A	N/A	53	14.3
Full	102	8.8	152	40.9
Unknown	27	2.3	37	10.0
*Inhalation injury*				
No	1132	97.2	197	53.0
Yes	33	2.8	175	47.0
*Intent*				
Unintentional	1139	97.8	274	73.7
Intentional	22	1.9	94	25.3
Unknown	4	0.3	4	1.1

#### Data analyses

In study I, adherence to each of the referral criteria (see Supplementary Figure 1) as well as adherence to the referral criteria (all aggregated) was measured as a proportion of cases in which at least one criterion was identified over the total number of cases. Association between individual referral criterion and patient care was measured using univariate logistic regression and expressed as Odds Ratios (ORs) with 95% confidence intervals (CIs). Patient care was defined as requiring surgery or hospital length of stay longer than 7 days. The analyses were stratified by patient age group: those <2 years and those ≥ 2 years.

In study II, associations between in-hospital mortality with patient (gender and age), injury (burn depth, size, inhalation, existing comorbidities and intent), and admission-related (referral status, time to admission and level of referring hospital) characteristics were assessed using univariate logistic regressions and expressed as ORs with 95% CIs. Patient-, injury-, and admission-related characteristics that were significantly associated with mortality were then entered into a multivariate logistic regression model with mortality as an outcome.

Observed mortality at the burns centre for each ABSI score was compared to the levels previous described [50].

### Remote diagnosis using image-based tools (Studies III and IV)

#### Image data collection

A database of acute burn images was established from two data sources: one data set comprising images obtained from several burns centres in South Africa, the other containing images obtained at the Uppsala Hospital burns centre in Sweden. Pictures were collected as part of an mHealth App for acute burns diagnosis (initially moBurnZA [68], then Vula (www.vulamobile.com)), or as part of care for wound closure follow-up. Pictures were collected in ‘real-life’ settings leading to varying quality, background, lighting, distance from the wound and using available camera devices, which included smartphones, and digital cameras. Together with the images, deidentified data related to the patient’s age group and gender, as well as injury information-related body part involved, burn mechanism and diagnosis (burn depth and size) determined by burn specialists were collected when available.

#### Remote consultation by specialists (Study III)

The third study consisted of the diagnostic evaluation of 51 burn images performed by 26 burns and emergency medicine specialists when using their own handheld device (smartphone or tablet). A total of 10 typical cases representing wounds most commonly seen at emergency centres in the Western Cape were identified and exemplified using five or six images for each case. These were then put together in an online questionnaire following the example previously published [70] using SurveyMonkey (SurveyMonkey Inc., San Mateo California USA, www.surveymonkey.com) which included a few background questions for the physicians, followed by the images (presented in random order) and for which physicians had to estimate the depth (on a scale of four) and the size (as percentage TBSA). The survey was sent out electronically to purposively selected participants based on their expertise to diagnose burns from three groups: a) South African physicians practicing as tele-expert in a local mHealth App (Vula [68];), b) other South African burns specialists who were in the professional network of participants from the first group, c) Swedish burns specialists who were involved in related studies [70]. The final group consisted of a) eleven South African emergency medicine specialists, b) eight South African burns specialists and c) seven Swedish burns specialists.

Diagnostic accuracy of the assessments of burn depth and size was measured using two-way mixed-effect intraclass correlation coefficient (ICC) with bedside clinical assessment performed by burns specialists as gold standard. Analyses were performed all cases aggregated, and stratified by case age group: paediatric and adult. The results were interpreted using the following definition [101]: ICC<0.70 signifies low correlation, 0.70 ≤ ICC≤0.80 signifies acceptable correlation, and ICC>0.80 signifies high correlation. For burn depth, sensitivity and specificity were also measured after dichotomizing the results on the surgical need of each burn.

#### Automated burn segmentation and surgery classification (Study IV)

The fourth study involved the training and assessment of two image-based deep-learning algorithms to a) identify and segment burn injuries from background and healthy skin b) classify burn depth dichotomized based on the surgical need for each burn.

A total of 1105 images presenting cleaned, acute burn wounds were included in this study. Patients with lighter Fitzpatrick skin types (1–3) [102] were collected in Sweden and represent 35% of the images (n = 391), while the other 65% of the images (n = 714) were collected in South Africa and consist of patients with darker Fitzpatrick skin types (4–6). A total of 536 background images obtained from publicly available online datasets [103,104] were also added to the training of the wound identifier algorithm in order to improve segmentation performances. Burn images were individually scaled according to available anthropomorphic measurements [105–107], and manually annotated on a pixel-by-pixel basis using a binary mask to segment the wound from healthy skin or background. Classification of the surgical need was performed on an image level based on the burn’s expert initial depth diagnosis.

Two deep CNNs were trained using a commercially available platform for medical image-based analyses Aiforia Create (Aiforia Create, Aiforia Hub, Helsinki, Finland). The first algorithm segments the burn wound from everything else in the image and was trained using 773 burn images (70% of the dataset) and the 536 background images. The training area included the whole image, and in burn images the area to be segmented was the burn area. The second algorithm classifies each image based on their depth into one of two categories: surgical burns that require skin grafting due to deep-partial or full thickness wounds; and non-surgical burns that are of superficial- or mid-partial thickness and are manageable with conservative treatment. Again, the same 70% of the dataset was used for training, and for this algorithm the wounds themselves were used as the training areas that were split into the two categories.

For both algorithms, testing was performed using 30% of the training set for three-fold cross-validation, and once optimal parameters were obtained, a final training using 100% of the training sets was performed prior to exporting and evaluating the algorithms on the validation set. Additional training and validations were performed stratified by skin types.

A maximum of 30,000 iterations were used for training, and the feature size was predefined at 125 and 190 units for the wound detection and surgery classification algorithms respectively. The following image augmentation settings were used for all algorithms: variation in scale (± 10%), aspect ratio (± 10%), shear distortion (± 10%), luminance (± 10%), contrast (± 10%), white balance (± 10%) and variation in image compression quality (40–60%).

For the wound identification algorithm, segmentation results were measured as sensitivity, precision and F1-score on a pixel level for each image and aggregated across respective image sets (for the training, and validation sets, and by skin types). Comparisons in the algorithms performances by skin type was assessed using a non-parametric Mann–Whitney U-test. For the surgery classification algorithm, an image was classified as a surgical burn when ≥1% of the wound’s pixels were identified as such. Success rate, sensitivity and specificity were measured with 95% CIs for the validation set overall and stratified by skin type.

## Results

### Evaluation of existing triage tools at specialized burns centres (Studies I and II)

#### Referral criteria (Study I)

Overall, 94.8% of the patients admitted to the two burns centres in the province (93.4% at Red Cross Hospital for paediatric cases, and 99.2% at Tygerberg Hospital for adult cases) were admitted in adherence with the local referral criteria. On average, 1.7 and 2.5 criteria were identified in paediatric and adult patients, respectively, who fulfilled at least one criterion. The criterion for anatomical site was often fulfilled in both paediatric and adult cases (observed in 85.2% and 93.8% of the patients respectively). Among paediatric patients, the age criterion (<2 years) was fulfilled by over half (51.9%) of the patients. All other criteria (severity 11.1%, inhalation injury 2.8%, existing comorbidity 3.0%, mechanism of injury 3.9% and severe associated injuries 0.0%) were all fulfilled by a small number of paediatric patients. In the paediatric population, there is a tendency of patients not fulfilling the criteria to have flame burns, of TBSA between 11% and 15% and of full thickness (data not shown [88],). At the adult burns centre, only three patients were admitted not in adherence with the referral criteria [89]. It is also possible that patients were admitted due to lack of transport availability or for other personal reasons.

Results of the univariate logistic regressions between referral criteria and intensive patient care (undergoing surgery or length of stay >7 days) at the paediatric burns centre are presented in Table 2. Adherence to the list of referral criteria as a whole was not associated with the care received when including all patients (OR = 0.96). However, patients not fulfilling the age criterion (≥2 years) had higher odds of intensive patient care than those who fulfilled that criterion (OR = 1.76). The criterion for severity was the only one significantly associated with patient care in patients of the younger age group, whereas that of inhalation injury was also significant in the older age group.

**Table 2.** Association between adherence to referral criteria and intensive patient care.

Criteria	Children <2 years old		Chidren ≥2 years old	
	Odds Ratios	95% CI	Odds Ratios	95% CI
Age	N/A	-	1.8	1.4–2.2
Anatomical site	1.4	0.8–2.3	1.2	0.7–1.8
Severity	19.4	8.6–43.9	11.6	5.6–23.8
Inhalation injury	2.2	0.7–6.9	5.5	1.8–16.6
Mechanism of injury	2.7	0.7–10.3	1.1	0.6–2.2
Existing comorbidity	1.8	0.7–4.5	0.5	0.2–1.4

#### Mortality prediction scores (Study II)

One in five of the patients admitted to the adults burns centre passed away during their stay, representing 76 patients. Association between mortality and patient, injury and admission-related characteristics was assessed for flame burn patients and results of the univariate and multivariate logistic regressions are presented in Table 3. Within patient characteristics, gender was associated with mortality whereas age was not. Regarding injury characteristics, burn depth and size as well as presence of inhalational component were all individually associated with mortality in the crude analyses. On the other hand, presence of an intentional component or of previous comorbidities did not affect the outcome. Regarding admission-related characteristics, referral status was the only variable that was individually associated with mortality whereby patients who were not referred from lower levels of care had 3.2 higher odds of a fatal injury than those who were referred. In the multivariate analyses, only gender, burn size and referral status remained significantly associated with higher odds of mortality after adjusting for all other variables.

**Table 3.** Univariate and multivariate associations between patient, injury and admission-related characteristics with in-hospital mortality for flame burn patients admitted at Tygerberg Hospital burns centre in 2015 and 2016 (n = 263).

			Mortality		Crude		Adjusted	
			n	%	Odds Ratios	95% CI	Odds Ratios	95% CI
Patient characteristics								
Gender								
	Men (n = 175)		39	22.3	Ref		Ref	
	Women (n = 88)		34	38.6	2.2	1.3–3.8	3.77	1.7–8.5
Injury characteristics								
Burn depth								
	Superficial or Mid Partial (n = 73)		7	9.6	Ref		Ref	
	Deep Partial or Full thickness (n = 164)		59	36.0	5.3	2.3–12.3	1.6	0.6–4.2
	No information (n = 26)		7	26.9	3.5	1.1–11.2	1.8	0.4–7.7
Burn size								
	By percentage increase TBSA (n = 263)		73	27.7	1.1	1.07–1.13	1.1	1.08–1.14
Inhalational injury								
	No (n = 99)		12	12.1	Ref		Ref	
	Yes (n = 164)		61	37.2	4.3	2.2–8.5	1.2	0.5–3.1
Admission-related characteristics								
Referral status								
	Referred (n = 223)		53	23.8	Ref		Ref	
	Not referred (n = 40)		20	50.0	3.2	1.6–6.4	2.8	1.1–7.4

When assessing the accuracy of the ABSI score predictions in the given patient population, we observed that the mean ABSI score was of six when considering all patients admitted at the centre, and of seven when considering only patients who sustained flame burns. Whereas the highest ABSI score for patients who did not sustain a flame burn was nine, in those who did, the ABSI score ranged from 2 to 13. For each ABSI score, the mortality observed was in the expected range. However, all patients in the highest risk group (with an ABSI score of 12 or 13) passed away during their stay.

### Remote diagnosis using image-based tools (Studies III and IV)

#### Remote consultation by specialists (Study III)

**Figure 1.**
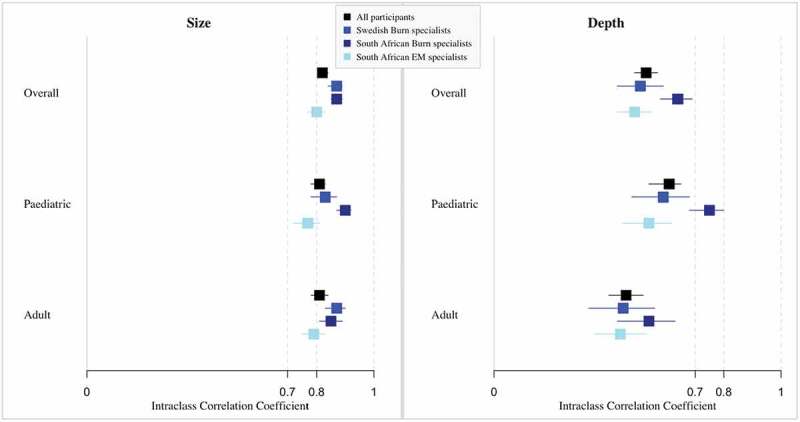
Diagnostic accuracy of size and depth assessments made on handheld devices by case and participant group.

The results of the diagnostic accuracy of the assessments of both burn size and depth performed by all 26 participants are presented in Figure 1. The assessments of burn size were of high accuracy for all participants, and for both paediatric and adult cases. Although still in the acceptable range, the assessments performed by South African Emergency Medicine specialists were slightly lower than those performed by burns specialists from both South Africa and Sweden. Assessments of burn depth were of relatively low accuracy even if assessments of paediatric cases were of higher accuracy than those of adult cases. In particular, the assessments of paediatric cases performed by South African burns specialists were considered of acceptable accuracy.

For size, 6 missing values, analysis performed on 1320 cases; for depth, 27 missing values, analysis performed on 1299 cases.

The sensitivity of the classification of burn depth according to the need for surgery was 75.6% when comparing the assessments performed on the handheld devices compared to the gold standard, and the specificity was 70.4%.

#### Automated burn segmentation and surgery classification (Study IV)

Training of the burn segmentation algorithm reached a sensitivity of 93.2% (95% CI: 92.4%–94.0%), and an F1 score of 90.2% (95% CI: 89.4%–91.0%). In the two trainings stratified by skin types, the sensitivity was 92.2% (95% CI: 90.9%–93.9%) and 93.4% (95% CI: 92.4%–94.4%) in lighter and darker skin types, respectively. In the complete validation set, the sensitivity was 86.9% (95% CI: 84.9%–89.0%), the precision 83.4% (95% CI: 81.5%–85.2%), and the F1 score was 82.9% (95% CI: 80.9%–84.9%). In the lighter skin types (Fitzpatrick 1–3), the sensitivity was 78.6% (95% CI: 74.0%–83.1%), and the F1 score was 76.9% (95% CI: 72.9%–80.9%) whereas the sensitivity and F1 scores for darker skin types (Fitzpatrick 4–6) were significantly higher (89.3% (95% CI: 87.2%–91.5%), and 87.8% (95%CI: 85.8%–89.7%) respectively; Mann–Whitney U-test *P* value = 0.0001).

**Figure 2.**
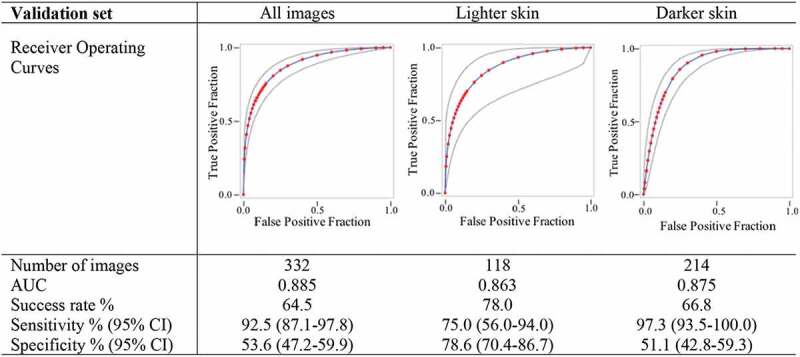
Wound classification algorithm performances results for the complete validation set as well as by skin type. Adapted from [108].

Regarding the surgical classification algorithm, the final training including the complete image set obtained a sensitivity of 99.6% and a specificity of 93.4%. In the stratified training sets, the sensitivity was 97.8% and 80.1% for lighter and darker skin types, respectively, and similarly the specificity was 100% and 97.1% in the two groups, respectively. The results of the validation sets, with all images and stratified by skin types are presented in Figure 2. The AUC was largest in the combined dataset with a value of 0.885. The overall sensitivity was 92.5% and the specificity was 53.6%. The sensitivity was highest for patients with darker skin types, whereas specificity was highest in patients with lighter skin types.

A total of 30 images included in the validation set had previously been assessed by the participants of Study III. The performances of the algorithm were of 71% and 30% for sensitivity and specificity respectively. As a comparison, the performances of the physicians were of 68% and 65% for the same images.

## Discussion

### Main findings

#### Triage protocols are followed at admission to the specialized burns centres

Differences in patient characteristics observed between the paediatric and the adult burns centres are in light with previous results from the African continent including the fact that children usually have smaller, less severe burns than adults do [109]. This is also reflected by the patient load between burns centres whereby the paediatric burns centre admits around 68 patients per bed annually versus 18 for the adults burns centre, and by the level of mortality that is extremely low at the paediatric burns centre [99].

Surprisingly, adherence to the referral criteria at admission to both burns centres was high. Indeed, high adherence is relatively rare at paediatric burns centres around the world where over-triage is commonly observed [23,40]. This difference could be explained by the criteria’s definition, the caseload, but also by local cultural practices and available resources. In particular, lack of bed availabilities, high transferring costs and lack of personal resources might all lead to higher rates of patients obtaining treatment at point-of-care in South Africa. This would thereafter lead to a very specific selection of patients being transferred to the specialized burns centres, independently of whether additional patients could have also been transferred. Indeed, previous results from lower levels of care in the Western Cape have shown that under-referral is high throughout the referral chain, from pre-hospital emergency services [30], to urban clinical health centres and district rural hospitals [110], and even at the trauma centres of specialised hospitals [34]. In the latest however, the patients who were adhering to the criteria but who were not referred tended to be very young patients (<2 years old) having particularly small burns, or those with a burn to a critical area [34]. The results obtained in this thesis show however that patients who are admitted to the specialised burns centres are correctly so [88]. Altogether, these results might put forward the needed distinction and existing discussion whereby referral criteria should be divided in order to specify which patients would benefit from immediate transfer to burns centres, and those who would require burns expertise in the form of remote consultation or outpatient treatment [25,39,69].

At the adult burns centre, mortality was associated – after adjustments for all other variables – with being a woman, having a large burn and being admitted without prior referral [89]. The mortality observed was in line with that seen across the African continent [111], and in another South African burns centre [112]. Regarding gender, more men were admitted to the burns centre, corroborating previous results that show men are more commonly transferred to higher levels of care in the country for a given severity [34,113,114]. Women were nonetheless more likely to succumb to their injury, confirming the presence of that variable in the ABSI score [50]. Possible explanations for this which have been put forward include: differences in physiological characteristics (body mass index and hormonal predispositions), as well as differences in treatment by health care professionals [50,113,115]. That burn size was associated with mortality was expectable. What was less expected however is that direct transfer to the burns centre was associated with increased mortality. An explanation could lie in the organisation of the local health system with pre-hospital emergency services bringing the most severe cases directly to the trauma centre of Tygerberg hospital knowing where the burns centre is located. These patients would have likely died prior to transfer if they had been cared for at lower levels of care [116].

The mortality observed at the burns centre was in line with that of the predictions estimated by the ABSI score. Nevertheless, an important difference to point out is that none of the patients in the highest ABSI risk group (with an ABSI score ≥12) survived their injury. This is informative for the health care system as those patients would likely have benefited from receiving palliative care closer to their relatives rather than being transferred and using the resources of the specialised centre.

#### Accurate diagnosis at point of care can be provided by digital health solutions

Image-based assessments performed on handheld devices were of high accuracy when considering burn size, and of lower accuracy when considering burn depth as compared to the gold standard. They were also overall of similar accuracy as when performed on a computer screen [70,90]. This confirms previous results in which lower accuracy for burn depth than for burn size is consistently observed when using photographic assessments [70,117,118]. These results support the notion that even these types of handheld devices could be used without providing less accurate standards, contrary to what the American Guidelines for Teleburn suggests [62]. Regarding the diagnosis of burn depth, it is of note that the results for South African burns specialists as well as those of paediatric cases were better than that of the other groups. The results of the surgical need classification were nonetheless acceptable, with higher rates than that found from untrained physicians.

The automated wound identification algorithm was of relatively high accuracy, with slightly better results than what had previously been obtained with similar deep-learning methods [85,86]. Results considering the surgery classification could be improved albeit the overall AUC of 0.885 is in the acceptable range, with similar results as those obtained in Study III. Differences were observed between cases of lighter and darker skin types, reflecting results previously obtained in remote consultation whereby skin-type characteristics have consequences on the diagnostic ability [70]. This also echoes a recent wound identification algorithm that showed lower accuracies for a combined model containing both African and Caucasian patients than separate models did [84]. It is of note that this method is at its start, and further work is required to obtain more specific depth classification, as well as to have some automated size calculation [119, 120]

### Methodological considerations

All four studies presented used different approaches for data collection and analyses. Each of these come with some methodological considerations which should be highlighted hereafter. Studies I and II used patient medical records that were individually retrieved and reviewed from the hospitals’ archives. Although regular data verification was performed, a number of patient files were either unidentifiable, unreachable, incomplete or unreadable. No systematic bias could be expected within these files, although it is possible that some of the paediatric patients with the most severe injuries were not retrieved due to ongoing judiciary follow-ups. Contrary to reports from lower levels of care in the province, most of the data was retrieved from the available files due to the patients’ rather long hospital stays. That being said, approximately 10% of the adult population had missing depth information. It is likely that the information was lacking when the depth did not impact on the outcome, when the burn was either very superficial (good outcome) or a very large burn (bad outcome). Several analyses were performed to verify this did not affect the obtained results. The other limitation associated with the retrospective data collection, is that both adherence to the referral criteria and to the ABSI score were evaluated on recorded information rather than as a prospective affirmation of reasons for admission. Furthermore, a difference in the time periods studied between the adult and the paediatric burns centres differed due to the difference in caseload between the units and to the aetiology of the cases at different age. Whereas only the most critical time of the year was studied at the paediatric centre (winter) was examined during five consecutive winters, it was decided to only look at two full calendar years at the adult hospital as injuries in that population are less affected by the season.

Study III relied on the use of an online survey and participants were recruited using a purposive sampling method, leading to potential sampling bias, and limiting the generalisability of the results. Given the small number of existing specialists, it is however unclear whether a different sample would have yield different results. This is even more so the case for sub-analyses when the number of specialists per group was small (n = 7, 8 and 12 for Swedish burns specialists, South African burns specialists and South African EM specialists respectively). This could therefore limit the interpretation of the results for the sub-groups, although the sample size was sufficient for the whole group given the relatively high number of images [101]. In order to increase participation and diminish participants fatigue, the survey was conducted in real-life settings with participants using their own devices, and their own pace, and the images were submitted in a random order. Nonetheless, a number of missing answers were recorded, and these could possibly represent the most complex cases in which further information or discussion between clinicians would have been useful.

Studies III and IV rely on the collection of a large image dataset. This is a unique asset which includes images collected from various sources, within and between countries, in order to cover as many possible situations. These images varied in background, lighting, colour and resolution, but also in demographic characteristics, body parts and sizes. In Study IV, the stand was taken to use all images that can be of clinical relevance rather than selecting images in order to improve the accuracy of the algorithm on a limited dataset. This does imply that higher accuracy levels might have been obtained if the images were more specifically selected, but this would have lower implementation possibilities in the long term. Ultimately, in both studies III and IV, images were used on their own for the diagnosis, whereas it is likely that when implemented other relevant clinical information would be provided as well as the possibility of close-to-live discussions with the clinician at point-of-care.

Finally, this thesis synthesis was constructed around four independent tools that are all relevant for decision-support, but the decision was made not to include them in a specific conceptual framework. The work was nonetheless freely inspired by the Mehrotra et al.’s conceptual framework for the referral process from primary to specialty care in the USA [121, 122].

## Conclusions

As a whole, the results of this work inform on four very different tools that individually can assist with the diagnosis, referral and triage of patients with acute burns. It provides a snapshot of the situation at admission to the burns centres in the Western Cape of South Africa, and prove the potential for digital health tools that are by now established or in development. These tools are all of relatively low costs, and could be even more powerful if used together as a simple addition to the current procedures. A simple example of how to implement these tools is to include triage checklists as part of the existing remote consultation mobile Apps. All referrals and admissions to burns centres would thereafter be conditional to the use of such an App, which could be beneficial for clinicians at point-of-care but also at receiving hospitals. The actual benefit of these tools on patients outcomes in the long term nonetheless remain to be assessed.

The implementation of all these methods have the aim to reduce inequity in access to appropriate healthcare by not only bringing specialists’ advices directly at bedside for all patients, but also by providing objective measures for decisions of patients’ referral to burns centres independent of any discriminations, time or location of admission. In a country like South Africa where inequalities are a major burden, any interventions to reduce differences between populations are truly beneficial.

The ultimate aim should be to provide the best care as possible for all patients. Until the available capacity and resources are limiting factors, triage decisions must be taken while aiming for the survival of the maximum number of patients in an objective manner, even if that results in denying treatment for the patients with very poor prognosis [5,17,20,47]. This does not only apply to resource-scarce settings, but also to higher income settings in times of disasters or crises [18,19]. They could therefore be used as an example for other situations where rapid and critical decisions need to be made such as in the current covid-19 pandemic where patients might outnumber the available intensive care bed availabilities.

A large portion of this work focuses on the South African context, but most of the results can also be applied in other settings, such as in Sweden where the reduction in burn injury prevalence has led to the over-centralisation of burn care, and to the lack of knowledge from clinicians at point-of-care. Remote consultation can therefore be a key asset, but also the inclusion of patients with different backgrounds and skin types in the algorithm training will permit its application worldwide. Nonetheless, automated diagnosis for burns care is only in its first steps and several improvements to the algorithms will be required prior to achieving sufficient healthcare-use accuracy. Further research will also be required in order to determine professional’s intention to use of such a technology, as well as the acceptance of patients to be treated with those novel methods.

## Author contribution

Single author Constance Boissin produced this PhD review based on her doctoral studies and thesis.

## Supplementary Material

Supplemental Material

## Paper context

Access to specialised care is costly and limited, especially in resource-scarce settings. Accurate diagnosis, triage and referral are all essential in order to maximise the benefits for both the patients and the healthcare system. This work focused on four clinical tools that are likely to assist, either at admission to burns centres for the triage of patients, or using the latest modern technologies to assist with diagnosis at point-of-care.
