# Supplementary material for: Clinical decision-support for acute burn referral and triage at specialized centres – Contribution from routine and digital health tools
Source: Glob Health Action. 2022 Jun 28;15(1):2067389. doi: 10.1080/16549716.2022.2067389 (PMC9246103; doi:10.1080/16549716.2022.2067389)
Supplement: Supplemental Material [file ZGHA_A_2067389_SM0573.docx]

**Age:** Less than 2 years or over 60 years.

**Severity:** Partial thickness burns with TBSA>15% in children or with TBSA >25% in adults; or full thickness burns with TBSA >15% in both children and adults.

**Anatomical site:** Face, hands, feet, genitalia, perineum, major joints, or circumferential burns. (These burns could also be dealt with at level 1 or 2 but discretion must be used).

**Inhalation injury:** Requiring ventilation for more than 48 hours.

**Mechanism of injury:** Exposure to ionizing radiation, high pressure steam, high tension electrical injury, hydrofluoric acid injury >1%, or suspicion of a non-accidental burn injury.

**Existing co-morbidity**: Cardiac limitation and/or myocardial infarction within 5 years, respiratory limitation of exercise, uncontrolled type 1 diabetes, pregnancy, medically or disease induced immune-suppression for any reason, existing psychiatric or suicidal tendencies, or suspected drug/alcohol abuse.

**Severe associated other injuries:** For example: polytrauma or crush syndrome.

**Supplementary Figure 1.** Western Cape provincial referral criteria for transfer to one of the two specialised burn centres [100].

**REFFERAL CRITERIA FOR TRANSFER TO BURN CENTRES**
